# Adjuvant β-Lactam Therapy Combined with Vancomycin or Daptomycin for Methicillin-Resistant Staphylococcus aureus Bacteremia: a Systematic Review and Meta-analysis

**DOI:** 10.1128/AAC.01377-20

**Published:** 2020-10-20

**Authors:** Chunjiang Wang, Chao Ye, Linglong Liao, Zhaohui Wang, Ying Hu, Chao Deng, Liang Liu

**Affiliations:** a Department of Pharmacy, The Third Xiangya Hospital, Central South University, Changsha, Hunan, China; b Department of Pharmacy, People’s Hospital of Ningxiang City, Hunan University of Chinese Medicine, Changsha, Hunan, China; c Gastroenterology, Zengcheng District People’s Hospital of Guangzhou, Guangzhou, Guangdong, China; d Department of Pharmacy, Jingshan Union Hospital of Huazhong University of Science and Technology, Jingshan, Hubei, China; e Neurosurgery, People’s Hospital of Ningxiang City, Hunan University of Chinese Medicine, Changsha, Hunan, China

**Keywords:** methicillin-resistant *Staphylococcus aureus*, bacteremia, β-lactams, vancomycin, daptomycin, combination therapy, meta-analysis

## Abstract

Infections due to methicillin-resistant Staphylococcus aureus bacteremia (MRSAB) seriously threaten public health due to poor outcomes and high mortality. The objective of this study is to perform a systematic review and meta-analysis of the current evidence on adjuvant β-lactam (BL) therapy combined with vancomycin (VAN) or daptomycin (DAP) for MRSAB. PubMed, Embase, and Cochrane Library were systematically searched for publications reporting clinical outcomes of BLs+VAN or BLs+DAP for adult patients with MRSAB through 5 April 2020. Meta-analysis techniques were applied using random effects modeling. Three randomized controlled trials and 12 retrospective cohort studies were identified, totaling 2,594 patients. Combination treatment significantly reduced the risk of clinical failure (risk ratio [RR] = 0.80; 95% confidence interval [CI], 0.66 to 0.96; *P* = 0.02; I^2^ = 39%), bacteremia recurrence (RR = 0.66; 95% CI, 0.50 to 0.86; *P* = 0.002; I^2^ = 0%), and persistent bacteremia (RR = 0.65; 95% CI, 0.55 to 0.76; *P* < 0.00001; I^2^ = 0%) and shortened the duration of bacteremia (standardized mean difference [SMD] = –0.37; 95% CI, –0.48 to –0.25; *P* < 0.00001; I^2^ = 0%). There was no significant difference in the risk of crude mortality, nephrotoxicity, or thrombocytopenia between groups. Notably, combination treatment might nonsignificantly increase the risk of Clostridium difficile infection (CDI) (RR = 2.13; 95% CI, 0.98 to 4.63; *P* = 0.06; I^2^ = 0%). Subgroup analysis suggested that DAP+BLs could reduce crude mortality (RR = 0.53; 95% CI, 0.28 to 0.98; *P* = 0.04; I^2^ = 0%). The meta-analysis suggested that although combination therapy with BLs could improve some microbial outcomes, it could not reduce crude mortality but might increase the risk of CDI and should be applied very cautiously. Regarding mortality reduction, the combination of DAP+cephalosporins appears more promising.

## Abstract (precis)

Infections due to methicillin-resistant Staphylococcus aureus bacteremia (MRSAB) seriously threaten public health due to poor outcomes and high mortality. The objective of this study is to perform a systematic review and meta-analysis of the current evidence on adjuvant β-lactam (BL) therapy combined with vancomycin (VAN) or daptomycin (DAP) for MRSAB. PubMed, Embase, and Cochrane Library were systematically searched for publications reporting clinical outcomes of BLs+VAN or BLs+DAP for adult patients with MRSAB through 5 April 2020.

## INTRODUCTION

Staphylococcus aureus is an important human pathogen and one of the leading causes of both nosocomial and community-acquired bacteremia worldwide. Staphylococcus aureus bacteremia (SAB) is a common cause of bloodstream infections, with an annual population-based incidence rate ranging from 20 to 30 cases/100,000 population in higher income countries (1). Even with adherence to current standards of care, all-cause mortality is still high. Case fatality rates for SAB remain stable between 15% and 50% (2). SAB carries a high risk of complications, such as endocarditis, septic shock, and disseminated infection (3), which are associated with a high risk of relapse and death from metastatic disease. Importantly, infection due to methicillin-resistant Staphylococcus aureus (MRSA) complicates therapy and has been identified as an independent risk factor for mortality (4, 5).

Vancomycin (VAN) and daptomycin (DAP) are the only agents currently approved for treating MRSA bacteremia (MRSAB) (5). However, each agent has limitations. Specifically, a number of issues hamper the utility of VAN, including slow bactericidal activity, low tissue penetration, and increasing reports of resistance and failure (6, 7). While DAP has been effective against MRSAB, unsusceptible isolates and treatment failures have been noted (8, 9).

Considerable efforts have been made to improve MRSAB treatment results and outcomes. Combinations of VAN or DAP with other antibacterial agents are being increasingly used to treat serious MRSA infections. Combination therapy with an active β-lactam (BL) early in the course of MRSAB has been suggested as a possible treatment strategy due to the observed synergy between glycopeptides and BLs (10–13). This phenomenon has been termed the ‘‘see-saw’’ effect, where, in the presence of glycopeptide or lipoglycopeptide, susceptibility to BLs improves (14–17). In recent years, an increasing number of studies have evaluated the effectiveness and safety of VAN or DAP combined with BLs in the treatment of MRSAB, especially at the beginning of 2020 (as of April 2020), and three clinical studies have been reported (18–20). However, the efficacy and safety of BLs as an adjuvant therapy for MRSAB are still ongoing matters of debate. Therefore, we decided to update the existing evidence to better determine the clinical effectiveness and safety of adjuvant BLs in the treatment of MRSAB with respect to crude mortality, nephrotoxicity, and Clostridium difficile infection (CDI), among others.

## RESULTS

### Identified studies.

**FIG 1:**
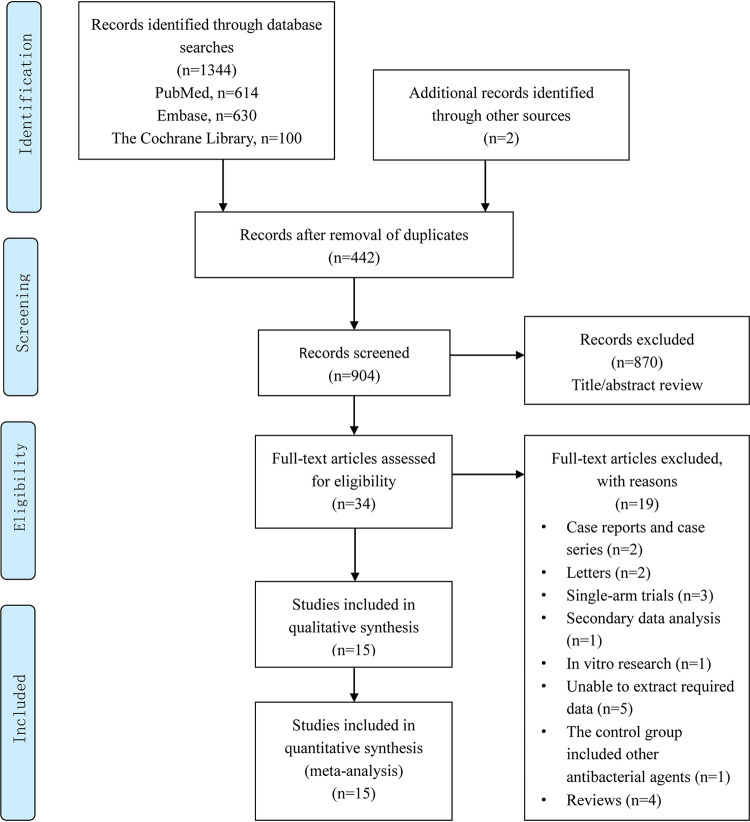
Flow diagram of the selection of studies for inclusion in the meta-analysis.

A total of 1,344 relevant studies were initially identified. After removing duplicate documents and screening the titles and abstracts, we determined that 34 studies were to be subject to a full-text assessment (Fig. 1). After applying the inclusion/exclusion criteria, a total of 15 studies (6, 18–31) comprising a total of 2,594 patients were included (1,189 patients in the standard therapy [STAN] group and 1,405 patients in the STAN therapy combined with β-lactams [COMBO] group), including 7 studies (21–27) based on the combination of VAN, 5 studies (6, 28–31) based on the combination of DAP, and 3 studies (18–20) based on the combination of daptomycin or vancomycin. Among the included studies, the β-lactam of choice was ceftaroline in four studies (20, 29–31), cefazolin in three studies (19, 23, 25), flucloxacillin in two studies (19, 22), cloxacillin in one study (19), and cefepime in one study (27). In six cohort studies, β-lactams generally comprised more than 3 antibacterial agents (18, 21, 26, 28) or unknown varieties (6, 24). Among the included studies, 3 were randomized controlled trials (RCTs) (19, 22, 30), 12 were retrospective cohort studies, 10 were multicenter studies, 4 were conducted at a single center, and 1 was unknown. Most of the studies were conducted in the United States, except for one RCT (22) in Australia and another four countries, namely, Australia, Singapore, Israel, and New Zealand (19). The main characteristics of the 15 included studies are shown in Table 1 and Table S2 in the supplemental material.

**TABLE 1:** Study characteristics

Author, yr (reference)	Country	Methodology	Study period (mo/year)	No. of centers	Age by treatment group (yrs)	APACHE II^*a*^ score by treatment group	Total no. of studies included	STAN^*b*^ group	COMBO^*c*^ group	Primary endpoints^*d*^	Safety endpoints^*d*^
Casapao, et al., 2017 (21)	USA	Retrospective cohort study	01/2010–12/2014	5	STAN：68; COMBO: 62	NR^*e*^	97	40 VAN^*f*^	57 VAN+BL^*g*^	1, 2, 3, 4, 5	6
Davis, et al., 2016 (22)	Australia	RCT	01/2010–05/2014	7	STAN：65; COMBO: 64	STAN：11; COMBO: 10.2	60	29 VAN	31 VAN+2 g q6h^*h*^ flucloxacillin	1, 3, 4, 5	Unable to extract
Jorgensen (1), et al., 2019 (23)	USA	Retrospective cohort study	2006–2019	Multicenter	STAN：51; COMBO: 52	STAN：13; COMBO: 18	237	133 VAN	104 VAN+cefazolin	1, 2, 3, 4	6
Taylor, et al., 2019 (24)	USA	Retrospective cohort study	01/2005–07/2017	NR	NR	NR	74	37 VAN	37 VAN+non-MRSA BL	2	NR
Trinh, et al., 2017 (25)	USA	Retrospective cohort study	01/2008–05/2017	1	STAN：58; COMBO: 51	STAN：13; COMBO: 11	101	60 VAN	41 VAN+cefazolin	1, 2, 3, 4	NR
Truong, et al., 2018 (26)	USA	Retrospective cohort study	01/2014–12/2016	1	STAN：57; COMBO: 62	STAN：16; COMBO: 21	110	47 VAN	63 VAN+BL^*i*^	1, 2, 3, 4, 5	6, 8
Zasowski, et al., 2019 (27)	USA	Retrospective cohort study	2006–2017	8	STAN：56; COMBO: 61	STAN：13; COMBO: 20	358	129 VAN	229 VAN+cefepime	1, 3, 4, 5	6, 7
Jorgensen (2), et al., 2019 (28)	USA	Retrospective cohort study	2008–2018	2	STAN：58; COMBO: 58	STAN：13; COMBO: 16	229	157 DAP^*j*^	72 DAP+ BL^*k*^	1, 2, 3, 4, 5	6, 7
Moise, et al., 2013 (6)	USA	Retrospective cohort study	2005–2009	Multicenter	NR	NR	56	34 DAP	22 DAP+ BL	2	NR
Alosaimy, et al., 2020 (18)	USA	Retrospective cohort study	2006–2019	8	STAN：57; COMBO: 59	STAN：14; COMBO: 19	597	153 VAN/DAP^*l*^	444 VAN/DAP^*m*^+BL^*n*^	1, 2, 3, 4, 5	6, 7, 8
Tong, et al., 2020 (19)	Australia, Singapore, Israel, New Zealand	RCT	08/2015–07/2018	27	STAN：63; COMBO: 65	NR	352	178 VAN/DAP^*o*^	174 VAN/DAP^*p*^+BL^*q*^	1, 2, 3, 4	6
Ahmad, et al., 2020 (20)	USA	Retrospective cohort study	01/2015–12/2017	1	STAN：41; COMBO: 46	NR	30	15 VAN/DAP	15 VAN/DAP+ceftaroline	1, 4	6
Fox, et al., 2018 (29)	USA	Retrospective cohort study	11/2010–03/2017	1	Unknown	NR	82	41 VAN	41 DAP+ceftaroline	2	NR
Geriak, et al., 2019 (30)	USA	RCT	01/2016–10/2017	3	STAN：62; COMBO: 62	NR	40	23 VAN(21)/DAP(2)	17 DAP+ceftaroline	1, 5	6
Mccreary, et al. 2019 (31)	USA	Retrospective cohort study	01/2013–10/2017	4	STAN：58; COMBO: 58	NR	171	113 VAN/DAP	58 DAP+ceftaroline	1, 4	NR

a APACHE II, Acute Physiology and Chronic Health Evaluation II.

b STAN, standard therapy.

c COMBO, standard therapy combined with β-lactams.

d Endpoints include the following: (1) crude mortality (calculated by including any relevant in-hospital mortality, 28/30-day mortality, 60-day mortality, or 90-day mortality); (2) clinical failure (composite endpoint); (3) persistent bacteremia (≥7 days or >5 days); (4) bacteremia relapse; (5) duration of bacteremia in days or hours (median, IQR); (6) nephrotoxicity; (7) Clostridium difficile infection; and (8) thrombocytopenia.

e NR, no report.

f VAN, vancomycin.

g BL, β-lactam antibiotic. Any one of the following agents: ampicillin, nafcillin, oxacillin, ampicillin/sulbactam, piperacillin-tazobactam, cefazolin, cefoxitin, ceftriaxone, ceftazidime, cefotaxime, cefepime, imipenem/cilastatin, doripenem, ertapenem, and meropenem.

h q6h, every 6 hours.

i Forty-three patients received piperacillin-tazobactam (34), ceftriaxone (4), ceftaroline (2), cefepime (2), or meropenem (1). Twenty patients received multiple BLs.

j DAP, daptomycin.

k The BLs included cefepime (43%), cefazolin (25%), ceftaroline (9.7%), ceftriaxone (9.7%), meropenem (9.7%), piperacillin-tazobactam (9.7%), ertapenem (1.4%), and ampicillin-sulbactam (1.4%).

l VAN only (54.2%), DAP only (6.5%), or VAN and DAP (39.2%).

m VAN only (58.1%), DAP only (2.5%), or VAN and DAP (39.4%).

n The BLs included cefepime (45.9%), cefazolin (33.6%), ceftaroline (12.2%), ceftriaxone (15.3%), piperacillin-tazobactam (15.3%), meropenem (6.3%), ampicillin-sulbactam (3.2%), and others (other carbapenems, monobactams, and cephalosporins; 1.6%).

o Received vancomycin (100%), daptomycin (3%).

p Received vancomycin (98%), daptomycin (4%).

q 2 g q6h flucloxacillin in Australia and New Zealand; 2 g q6h cloxacillin in Singapore and Israel; 111 patients received only flucloxacillin or cloxacillin, and 27 received only cefazolin.

### Quality assessment.

The quality of the 12 included cohort studies was evaluated using the Newcastle-Ottawa scale (NOS). There were a total of seven studies with NOS scores of ≥6 points, of which three scored 7 points (18, 26, 28) and four scored 6 points (20, 21, 27, 31). Studies published in the abstract form all scored <6 points, of which three (23–25) scored 3 points and one (29) scored 4 points. Another study (6) scored 5 points because it was not scored in comparability (see Table S3 in the supplemental material). The Cochrane Collaboration “risk of bias” tool was used to assess the quality of the RCTs. All included studies were registered with clinical trial registration numbers. For the Tong study (19), seven evaluation indicators were all low risk. For the Davis study (22), although the risks of performance bias and detection bias were unclear, the other five evaluation parameters were all low risk. For the Geriak study (30), excluding detection bias, attribution bias, and reporting bias, which were low risk, the risk assessment of the other four indicators was unclear (see Fig. S1 in the supplemental material).

### Crude mortality.

**FIG 2:**
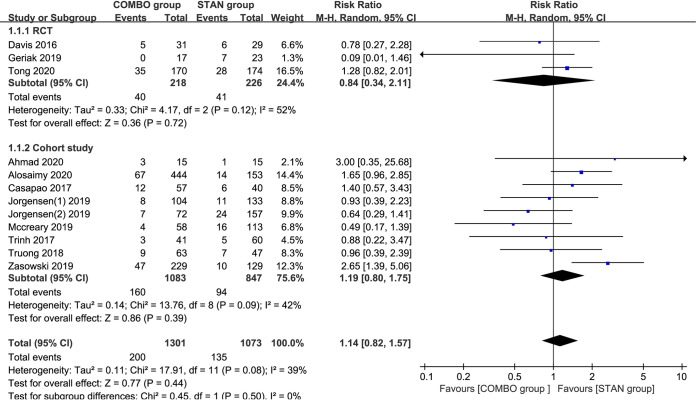
Forest plot of the risk ratio (RR) for crude mortality in patients with MRSA bacteremia.

A meta-analysis of the 12 studies (18–23, 25–28, 30, 31) including 2,374 patients suggested that there was no significant difference in the rate of crude mortality between the COMBO and STAN groups (risk ratio [RR] = 1.14; 95% confidence interval [CI], 0.82 to 1.57; *P* = 0.44; I^2^ = 39%) (Fig. 2). By excluding 2 cohort studies (23, 25) with scores of <6 points, the meta-analysis results did not change significantly (RR = 1.16; 95% CI, 0.80 to 1.68; *P* = 0.45; I^2^ = 48%). However, a subgroup analysis of three studies (440 patients in total) (28, 30, 31) showed that the combination of DAP with BLs could reduce the risk of crude mortality (RR = 0.53; 95% CI, 0.28 to 0.98; *P* = 0.04; I^2^ = 0%) (Table 2). The results of the other subgroup analyses were similar to the overall meta-analysis results (Table 2).

**TABLE 2:** Subgroup analysis results of different outcome indicators

Outcome (subjects)	Subgroup^*a*^	No. of studies	RR^*b*^ (95% CI)	*P* value	I^2^ (%)
Crude mortality	VAN+BL	6	1.28 (0.83–1.99)	0.26	27
	DAP+BL	3	0.53 (0.28–0.98)	0.04	0
	BL (ceftaroline)	3	0.58 (0.12–2.83)	0.5	52
	RCTs	3	0.84 (0.34–2.11)	0.72	52
	Cohort studies	9	1.19 (0.80–1.75)	0.39	42
Clinical failure	VAN+BL	6	0.79 (0.59–1.06）	0.11	55
	DAP+BL	4	0.75 (0.46–1.22)	0.25	23
	BL (ceftaroline)	1	0.93 (0.52–1.68)	0.82	NA^*c*^
	RCT	1	0.89 (0.68–1.18)	0.43	NA
	Cohort studies	9	0.77 (0.62–0.97)	0.02	44
Bacteremia recurrence	VAN+BL	6	0.61 (0.39–0.96)	0.03	0
	DAP+BL	2	0.72 (0.39–1.35)	0.31	0
	BL=Ceftaroline	2	0.81 (0.31–2.11)	0.66	0
	RCTs	2	0.77 (0.40–1.48)	0.44	0
	Cohort studies	9	0.63 (0.47–0.85)	0.002	0
Persistent bacteremia	VAN+BL	6	0.61 (0.47–0.79)	0.0002	0
	DAP+BL	1	0.74 (0.43–1.28)	0.28	NA
	RCTs	2	0.54 (0.32–0.88)	0.01	0
	Cohort studies	7	0.66 (0.56–0.79)	<0.00001	0
Nephrotoxicity	VAN+BL	4	0.93 (0.55–1.59)	0.8	18
	DAP+BL	2	2.11 (0.33–13.47)	0.43	34
	BL (ceftaroline)	1	0.44 (0.02–10.29)	0.61	NA
	RCTs	2	2.29 (0.39–13.53)	0.36	41
	Cohort studies	7	1.08 (0.75–1.54)	0.68	21

a VAN, vancomycin; DAP, daptomycin; BL, β-lactam antibiotic.

b RR, risk ratio.

c NA, not applicable.

### Clinical failure.

**FIG 3:**
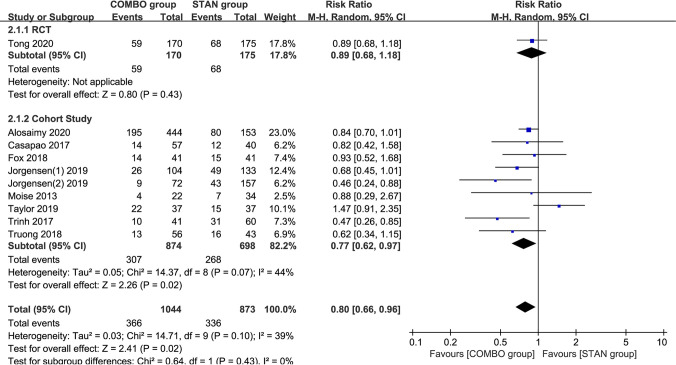
Forest plot of the risk ratio (RR) for clinical failure in patients with MRSA bacteremia.

The results of the meta-analysis of 10 studies (a total of 1,917 patients) (6, 18, 19, 21, 23–26, 28, 29) suggested that COMBO therapy could significantly reduce the risk of clinical failure (RR = 0.80; 95% CI, 0.66 to 0.96; *P* = 0.02; I^2^ = 39%) (Fig. 3). By excluding five cohort studies (6, 23–25, 29) with scores of <6 points, the meta-analysis results did not change significantly (RR = 0.81; 95% CI, 0.69 to 0.95; *P* = 0.008; I^2^ = 7%). A subgroup analysis indicated that, except for the results of the meta-analysis of the cohort studies, similar results were obtained (RR = 0.77; 95% CI, 0.62 to 0.97; *P* = 0.02; I^2^ = 44%), and no significant differences were found in the other subgroups (Table 2).

### Bacteremia recurrence.

**FIG 4:**
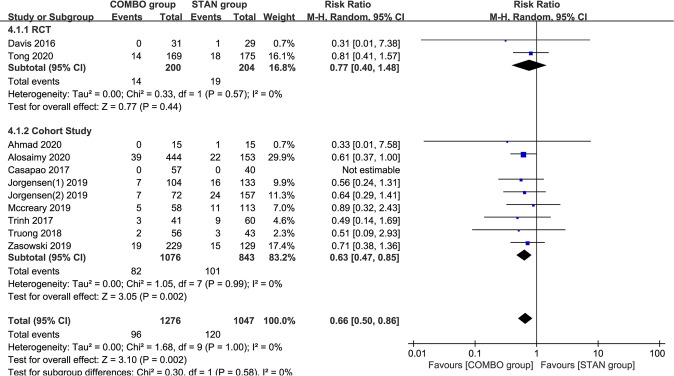
Forest plot of the risk ratio (RR) for bacteremia recurrence in patients with MRSA bacteremia.

Eleven studies (a total of 2,323 patients) reported bacteremia recurrence (18–23, 25–28, 31). The meta-analysis results showed that COMBO therapy could significantly reduce the risk of bacteremia recurrence (RR = 0.66; 95% CI, 0.50 to 0.86; *P* = 0.002; I^2^ = 0%) (Fig. 4). After we excluded two cohort studies (23, 25) with scores of <6 points, the meta-analysis results did not change significantly (RR = 0.68; 95% CI, 0.51 to 0.90; *P* = 0.008; I^2^ = 0%). Subgroup analysis suggested that the results of the meta-analysis of VAN combined with BLs (RR = 0.61; 95% CI, 0.39 to 0.96; *P* = 0.03; I^2^ = 0%) and cohort studies (RR = 0.63; 95% CI, 0.47 to 0.85; *P* = 0.002; I^2^ = 0%) yielded similar results (Table 2). However, other subgroup analyses suggest that COMBO treatment did not significantly reduce the risk of bacteremia recurrence (Table 2).

### Duration of bacteremia.

**FIG 5:**
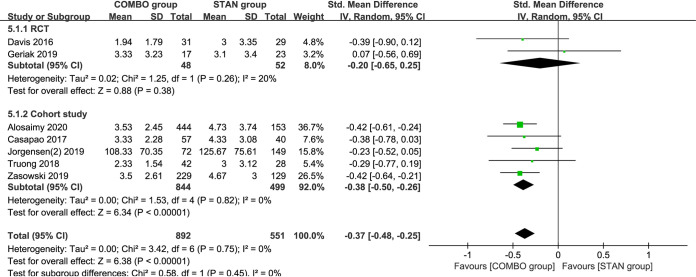
Forest plot of the standardized mean difference (SMD) for the duration of bacteremia in patients with MRSA bacteremia.

A meta-analysis of seven studies (18, 21, 22, 26–28, 30) including 1,443 patients suggested that COMBO treatment could significantly shorten the duration of bacteremia (standardized mean difference [SMD] = –0.37; 95% CI, –0.48 to –0.25; *P* < 0.00001; I^2^ = 0%) (Fig. 5). Subgroup analysis suggested that the results of the meta-analysis of VAN combined with BLs (SMD = –0.40; 95% CI, –0.56 to –0.23; *P* < 0.00001; I^2^ = 0%) and cohort studies (SMD = –0.38; 95% CI, –0.50 to –0.26; *P* < 0.00001; I^2^ = 0%) yielded similar results (Table 3).

**TABLE 3:** Subgroup analysis results of the duration of bacteremia

Outcome (subjects)	Subgroup^*a*^	No. of studies	SMD^*b*^ (95% CI)	*P* value	I^2^ (%)
Duration of bacteremia	VAN+BL	4	–0.40 (–0.56 to –0.23)	<0.00001	0
	DAP+BL	2	–0.18 (–0.44 to 0.07)	0.16	0
	BL (ceftaroline)	1	0.07 (–0.56 to 0.69)	0.83	NA^*c*^
	RCTs	2	–0.2 (–0.65 to 0.25)	0.38	20
	Cohort studies	5	–0.38 (–0.50 to –0.26)	<0.00001	0

a VAN, vancomycin; DAP, daptomycin; BL, β-lactam antibiotic.

b SMD, standardized mean difference.

c NA, not applicable.

### Persistent bacteremia.

**FIG 6:**
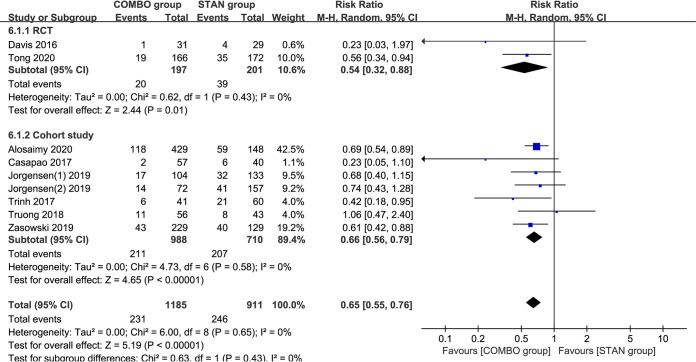
Forest plot of the risk ratio (RR) for persistent bacteremia in patients with MRSA bacteremia.

A meta-analysis of nine studies (18, 19, 21–23, 25–28) including 2,096 patients suggested that COMBO therapy could significantly reduce the risk of persistent bacteremia (RR = 0.65; 95% CI, 0.55 to 0.76; *P* < 0.00001; I^2^ = 0%) (Fig. 6). The results of the subgroup analysis suggested that VAN combined with BLs could significantly reduce the incidence of persistent bacteremia (RR = 0.61; 95% CI, 0.47 to 0.79; *P* = 0.0002; I^2^ = 0%) (Table 2), and the results of the subgroup analysis of different study types also provided similar conclusions (for RCTs: RR = 0.54; 95% CI, 0.32 to 0.88; *P* = 0.01; I^2^ = 0%; for cohort studies: RR = 0.66; 95% CI, 0.56 to 0.79; *P* < 0.00001; I^2^ = 0%) (Table 2).

### Adverse reactions.

**FIG 7:**
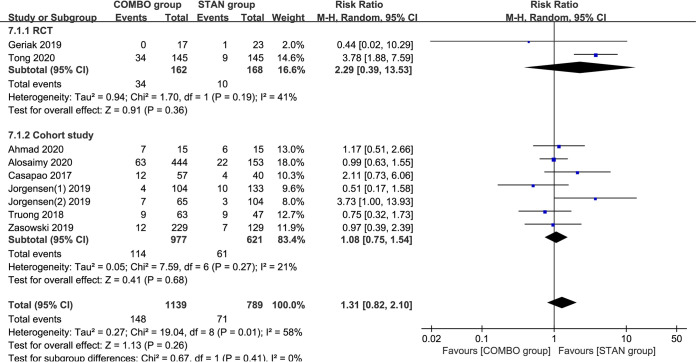
Forest plot of the risk ratio (RR) for nephrotoxicity in patients with MRSA bacteremia.

A meta-analysis of nine studies (18–21, 23, 26–28, 30) including 1,928 patients suggested that COMBO therapy did not significantly increase the risk of nephrotoxicity (RR = 1.31; 95% CI, 0.82 to 2.10; *P* = 0.26; I^2^ = 58%) (Fig. 7). After we excluded one cohort study (23) with scores of <6 points, the meta-analysis results did not change significantly (RR = 1.45; 95% CI, 0.89 to 2.35; *P* = 0.13; I^2^ = 57%). The results of the different subgroup analyses were similar to the overall meta-analysis results (Table 2).

A meta-analysis of three studies (18, 27, 28) including 1,184 patients suggested that COMBO therapy might nonsignificantly increase the risk of CDI (RR = 2.13; 95% CI, 0.98 to 4.63; *P* = 0.06; I^2^ = 0%) (see Fig. S2 in the supplemental material).

A meta-analysis of two studies (18, 26) including 707 patients demonstrated that COMBO therapy did not significantly increase the risk of thrombocytopenia (RR = 1.20; 95% CI, 0.78 to 1.85; *P* = 0.41; I^2^ = 0%) (see Fig. S3 in the supplemental material).

### Publication bias.

The funnel chart for the main outcome indicator of crude mortality and the publication bias test revealed a basically symmetrical left and right side of the funnel chart, and combined with Egger’s test results (bias, –1.42; 95% CI, –3.26 to 0.42; *P* = 0.116), these findings suggested a small possibility of publication (see Fig. S4 in the supplemental material).

## DISCUSSION

The main finding of this meta-analysis of 2,594 patients with MRSAB was the absence of statistically significant differences in the risk of crude mortality between the COMBO and STAN treatments. The COMBO treatment showed obvious advantages over the STAN treatment in reducing clinical failure, bacteremia recurrence, and persistent bacteremia and in shortening the duration of bacteremia outcome indicators. However, it should be noted that although nephrotoxicity and thrombocytopenia were not significantly different between the two groups, COMBO treatment may increase the risk of CDI.

The promise of the efficacy of combination therapy for SAB demonstrated using *in vitro* and animal models was not borne out in our meta-analysis measuring the primary outcome of crude mortality. At present, attempts to adopt a combined treatment regimen for SAB have not yielded positive results. Neither the addition of an aminoglycoside (32) for S. aureus endocarditis nor rifampicin (33, 34) for SAB resulted in improved clinical outcomes. However, it is encouraging that, in our study, the results of the subgroup analysis suggested that DAP combined with BLs significantly reduced the risk of crude mortality (RR = 0.53; 95% CI, 0.28 to 0.98; *P* = 0.04; I^2^ = 0%). The above encouraging results included a total of 3 studies including 430 patients (28, 30, 31), of which 1 was a non-double blind RCT study (30). This small-scale study of 40 patients showed that the initial treatment of DAP+ceftaroline might be associated with a reduction of in-hospital mortality compared with VAN or DAP monotherapy for patients with MRSAB (COMBO, 0% [0/17]; STAN, 26% [6/23]; *P* = 0.029). The results of the meta-analysis of the other two cohort studies (28, 31) suggested that DAP combined with BLs had a tendency to reduce mortality, although the results were not statistically significant (RR = 0.58; 95% CI, 0.31 to 1.09; *P* = 0.09; I^2^ = 0%). To minimize the effects of bias and confounding variables, the two cohort studies used propensity score matching (28, 31). In addition, I^2^ = 0 indicates less heterogeneity; thus, the results of the subgroup meta-analysis are relatively reliable.

For the results of the clinical benefit of crude mortality, we further analyzed the relevant factors that might affect the results. We found that the BLs in these three studies were almost all cephalosporins (two studies were ceftaroline [30, 31] and one study was mainly cefepime and cefazolin [28]), which seems to indicate that cephalosporin, especially ceftaroline, is a better choice as an adjuvant. This assertion appears to be explained by the following: ceftaroline itself has anti-MRSA activity, and *in vitro* studies have shown that ceftaroline offers dual benefit via synergy with both daptomycin and bolsters the innate immune response to attenuate the virulence of the pathogen (35). However, the subgroup analysis results of ceftaroline as an adjuvant β-lactam (20, 30, 31) do not support the above speculation (RR = 0.58; 95% CI, 0.12 to 2.83; *P* = 0.5; I^2^ = 52%). The three studies all used DAP or VAN combined with ceftaroline as an intervention therapy, but the combined effect size had a certain heterogeneity, which might be due to clinical heterogeneity (such as the severity of disease and different comorbidities) and methodological heterogeneity (such as different timings of administration). In the Ahmed (20), all evaluable patients had bacteremia lasting 4 days or longer after standard treatment, accompanied by natural valve infective endocarditis, osteomyelitis, and/or brain abscesses, and treatments containing ceftaroline were used as salvage treatments. However, the patients in the Geriak study (30) were not all refractory or complicated infections, and combination therapy was administered at the beginning. McCreary et al.(31) performed an exploratory analysis of the treatment of MRSAB with DAP+ceftaroline and STAN treatment. Subgroup analysis showed that within 72 hours of index culture, the mortality of patients in the COMBO group was significantly reduced. The Jorgensen study (28) provides evidence that DAP+BL is of benefit when used earlier in the infection course, before prolonged exposure to antibiotics and host cationic antimicrobial peptides have established the “perfect storm” for refractory infection. Therefore, the main reason for the difference in the above results may be that the timing of administration is different. We speculate that early combined therapy may provide the presumed clinical benefit, and if the combined regimen is used as a rescue treatment for refractory infections, the clinical benefit will be mitigated.

From the perspective of safety, cephalosporins are also more suitable β-lactam adjuvants than antistaphylococcal penicillins (ASPs). Although the overall results of our meta-analysis suggest that COMBO treatment does not significantly increase the occurrence of nephrotoxicity (RR = 1.31; 95% CI, 0.82 to 2.10; *P* = 0.26; I^2^ = 58%), the existence of wide heterogeneity affects their reliability. As evident in the forest plot (Fig. 7), two studies may be the main cause of the heterogeneity (19, 28). The CAMERA 2 study (19) was discontinued due to an increased risk of nephrotoxicity associated with the combined treatment regimen containing ASPs. The CAMERA 2 study was an open-label multicenter, randomized, controlled trial that enrolled 352 patients with MRSAB at 27 centers in 4 countries. Compared with STAN treatment, the 90-day all-cause mortality (21% [35/170] versus 16% [28/174]; *P* = 0.28) and acute kidney injury (AKI) incidence (23% [34/145] versus 6% [9/145]; *P* < 0.001) were numerically higher in patients receiving the COMBO treatment. Among those with AKI, nephrotoxicity incidence varied substantially between those treated with only flucloxacillin/cloxacillin (27% [30/111]) and only cefazolin (4% [1/27]). The concept that the degree of nephrotoxicity is drug specific has been supported in previous research. A meta-analysis assessing the risk of AKI with VAN combined with piperacillin-tazobactam suggested that the combination of VAN plus piperacillin-tazobactam increased the odds of AKI compared with that of VAN monotherapy, VAN plus cefepime or carbapenem, and piperacillin-tazobactam monotherapy (36). Moreover, a systematic review and meta-analysis to evaluate the safety of cefazolin and ASPs suggested that compared with ASPs, cefazolin was associated with significant reductions in nephrotoxicity in hospitalized patients and outpatients (hospitalized patients, Peto odds ratio [OR] = 0.225; 95% CI, 0.127 to 0.513; *P* < 0.001; outpatients, Peto OR = 0.372; 95% CI, 0.192 to 0.722; *P* = 0.003) (37). In the meta-analysis, nephrotoxicity was the primary endpoint, with several subgroups based on the nephrotoxicity definition and publication status, and acute interstitial nephritis was identified as the cause of nephrotoxicity. Notably, none of the patients on cefazolin had acute interstitial nephritis, compared with 8.82% of patients on ASPs (Peto OR = 0.189; 95% CI, 0.053 to 0.675; *P* = 0.010). In our included studies, although there were two RCT studies (19, 22) in which BLs were ASPs, only the CAMERA 2 nephrotoxicity data could be extracted, precluding a meta-analysis. However, combined with current evidence, to reduce the increased risk of nephrotoxicity in the combined regimen, the use of VAN combined with an ASP treatment regimen should be avoided. Unexpectedly, the Jorgensen study (28) also showed that patients with DAP combined with BLs had higher AKI. BLs in this study were mainly cephalosporins, making this safety signal particularly perplexing. However, the most experienced AKI patients received at least one concomitant nephrotoxin (for example, intravenous [i.v.] contrast dye, vancomycin, diuretic) within 72 hours before AKI. Therefore, minimizing exposure to concomitant nephrotoxins is an important consideration when utilizing DAP+BL. In addition, the risk of additive adverse effects should prompt careful consideration of patient selection before clinical application of combination therapy.

Our meta-analysis results raise another very important safety issue; COMBO therapy may increase the risk of CDI, although the difference between the two groups was not statistically significant (RR = 2.13; 95% CI, 0.98 to 4.63; *P* = 0.06; I^2^ = 0%). Many previous studies have also observed this phenomenon. Hung et al. (38) conducted a retrospective analysis to study the significance of toxigenic Clostridium difficile colonization (tCDC) in hospitalized patients and found that compared with monotherapy, patients were more likely to have tCDC if they received more than one antibiotic treatment (odds ratio [OR] = 6.67; 95% CI, 1.41 to 31.56; *P* = 0.01), particularly if they received a glycopeptide in combination with a cephalosporin or penicillin or a cephalosporin and a carbapenem combination, which was associated with a higher CDI incidence than that of a monotherapy. Another retrospective cohort study (39) also found that the incidence of CDI increased with the number of antimicrobials administered (RR = 2.01; 95% CI, 1.67 to 2.40), providing a reminder that combination therapy is associated with collateral damage and must be used judiciously.

A recent meta-analysis (published after completion of our work) compared the clinical efficacy and safety of the combination of VAN or DAP with BL versus VAN or DAP monotherapy in MRSA bacteremia or endocarditis (40). The conclusion of that meta-analysis is consistent with our conclusion that COMBO therapy has a lower risk of clinical failure (OR = 0.56; 95% CI, 0.39 to 0.79; *P* = 0.001; I^2^ = 26.22%), but there was no significant difference between the two treatment options in terms of mortality and nephrotoxicity. Nevertheless, the main difference is that we identified more studies (15 versus 9 studies) and, more importantly, more patients than those reported in reference 40 (2,594 versus 1,636 patients). This difference may stem from, in terms of search dates, a deadline for our meta-analysis of April and that of reference 40 of February. In addition, our meta-analysis included 6 more observational studies (6, 18, 20, 24, 25, 29) than the previously published meta-analysis because more outcome indicators were evaluated, and the inclusion and exclusion of studies were more rigorous and reasonable. Furthermore, the effect size in our meta-analysis was determined using the RR, which is easier to understand than the OR used in the meta-analysis of reference 40. More importantly, due to the large heterogeneity between observational studies and RCTs, which may have a greater impact on the results, we conducted an independent analysis of the data according to the type of studies; the meta-analysis in reference 40 combined RCTs with observational studies, which is inappropriate.

Some limitations should be considered when interpreting the results of this analysis. First, although we included more studies than previous meta-analyses, we did not include unpublished conference papers in the search. Second, to evaluate β-lactam more completely and realistically as an adjunct treatment method for MRSAB, we included different types of studies and different combinations of BL, which revealed obvious heterogeneity. Therefore, a random effects model was used for data aggregation between different study types. Concurrently, we conducted a subgroup analysis of different study types and different treatment combinations to evaluate whether study outcomes could be influenced by specific factors or subpopulations. In addition, because the inclusion of conference articles might affect the results of the combined effect results, we conducted a sensitivity analysis of different outcome indicators, deleted the study with an NOS score of <6 points, and evaluated the stability of the results. After the deletion of low-quality literature, there was no significant change in the data for combined effects. Third, a limitation of our analysis includes the retrospective nature of 12 of the 15 studies. Retrospective studies have a high selection bias and may expose the analysis to confounders. In general, the choice of combination therapy versus monotherapy was based on the physician’s discretion. As such, in most cases, critically ill patients in cohort studies tend to have a higher risk of death and are more likely to receive COMBO therapy. For example, among the 6 cohort studies that obtained an APACHE II score, the patients in the combined treatment group had a higher APACHE II score in 5 studies (18, 23, 26–28). The clinical effect of COMBO therapy may be weakened due to the severity of the disease. For the same reason, if COMBO therapy is used to rescue patients with persistent bacteremia after STAN treatment, the clinical effect of the combination may also be reduced. The McCreary study (31) suggested that the 30-day mortality rate of the patients treated with DAP+ceftaroline within 72 hours of blood culture was significantly lower than that of the standard treatment group (8.3% versus 14.2%; *P* > 0.05). However, in the Ahmad study (20), salvage therapy of the ceftaroline combination regimen did not yield a positive result (mortality rate of 20% in the COMBO group and 7% in the STAN group). Although combination therapy is mainly carried out in the early stage in most studies, some studies continue to include rescue therapy to evaluate the clinical efficacy. Finally, due to the inability to extract data, we could not analyze the effects of related factors, such as drug dosage, treatment duration, treatment timing, infection site, source control, and pathogen resistance, on the results, but a comprehensive consideration must be performed to choose the most suitable treatment plan.

In conclusion, the current meta-analysis showed that although COMBO therapy could reduce clinical failure, the recurrence of bacteremia, persistent bacteremia, and the duration of bacteremia, BLs as adjuvant therapy for MRSA bacteremia could not reduce crude mortality compared with STAN treatment. In addition, although our analysis results suggest that the two groups have no significant differences in safety outcomes, such as nephrotoxicity, CDI, and thrombocytopenia, COMBO treatment may increase the risk of CDI, and a specific combination may increase the risk of nephrotoxicity. Therefore, it is important to consider the risk-benefit of adding a second antimicrobial agent for the management of MRSAB. Since most of the included studies were cohort studies, the results may be challenged by inherent limitations and unmeasured confounding factors related to the design of this study. In the future, more randomized controlled studies are needed to focus on combination therapy combinations, doses, administration methods, and duration of treatment for assessing the evidence for the mortality and safety of combination therapy.

## MATERIALS AND METHODS

### Information sources and key word search.

Using the PubMed, Embase, and Cochrane databases, searches for relevant articles were performed with the following items: “(daptomycin or vancomycin or) and (methicillin-resistant Staphylococcus aureus or MRSA) and (bacteremia or septicemia or bloodstream infection).” Searches were limited to articles published in English up to 5 April 2020. In addition, the reference lists of reports identified by this search strategy were also searched to select relevant articles. The review protocol was registered at the Prospero international prospective register of systematic reviews (registration no. CRD42020175124).

### Inclusion and exclusion criteria.

The related literature was evaluated by reviewing the titles and abstracts and was further assessed by reviewing the full texts. Studies involving adult patients with MRSAB were included. Participants received two types of therapy, namely, standard therapy (STAN treatment; VAN or DAP alone treatment) and standard therapy combined with β-lactams (COMBO treatment); otherwise, the treatments were regarded as ineligible. BLs are antibacterial agents with antistaphylococcal activity, which are divided into penicillins, cephalosporins, and carbapenems. We required one or more of the following outcomes to be reported by the authors: (i) primary outcome of crude mortality (since mortality endpoints were different across studies, a composite outcome—defined as crude mortality—was also calculated by including any relevant comparison of mortality rates between STAN and COMBO treatment, irrespective of the definition used [i.e., all-cause, in-hospital, 30-day, 60-day, and 90-day mortality]); when data for more than one endpoint were available, mortality in the main analysis was recorded at the latest point in the study (e.g., 90-day mortality had precedence over 30-day mortality); (ii) secondary outcomes of clinical failure (composite endpoint) (Table S1), persistent bacteremia (≥7 days or >5 days), bacteremia recurrence, and duration of bacteremia in days or hours (median, interquartile range [IQR]); and (iii) safety outcomes of nephrotoxicity (Table S1), CDI, and thrombocytopenia. The following criteria were used to exclude studies from the analysis: republished literature containing only the latest and most comprehensive data, case reports and case series, and incomplete study data making it impossible to obtain the required data.

### Data extraction and quality assessment.

Two independent authors (C.W. and Z.W.) screened the titles and abstracts of records to evaluate potentially eligible articles. After an initial screening, all full-text articles were reviewed independently for inclusion eligibility. Discrepancies were resolved by consensus. If there was no agreement, a third author decided whether the article should be included (C.Y.). Data were extracted from the included studies independently by two authors (C.D. and L.L.) and were standardized using a data extraction table. Variables of interest included the number of patients included in each group, publication year, study design, location, enrollment period, patient characteristics (age, disease severity, and the source of infection), intervention and comparison, and outcome measures. A study-level risk of bias was assessed by two investigators (Y.H. and L.L.), with discrepancies resolved by a third investigator (C.Y.). The risk of bias of the included randomized controlled trials (RCTs) was assessed using the Cochrane risk of bias tool (41). When observational studies were considered, the Newcastle-Ottawa Scale (NOS) was used to assess the risk of bias in patient selection, comparability between groups, and outcome and exposure factor assessment (42). NOS scores range from 0 to 9, with scores ≥6 indicating good quality (43, 44).

### Statistical analysis.

For statistical analysis, a meta-analysis was performed using Review Manager software (version 5.3) to produce forest, an assessment of heterogeneity, and summary effect estimates. For dichotomous outcomes, we calculated the risk ratio (RR) and 95% confidence interval (CI). Continuous outcomes are presented as the standardized mean difference (SMD) with 95% CI. A random effects model was used to pool data due to the large heterogeneity between cohort studies (45). Forest plots were generated, and study heterogeneity was investigated using the I^2^ statistic (46). The heterogeneity of the included studies was assessed using the I^2^ statistic, with an I^2^ of >50% indicating a substantial level of heterogeneity (46). Subgroup analysis was conducted for different study types (for example, RCTs and cohort studies) and different combination treatment groups (such as VAN- and DAP-based combination and BL as ceftaroline). Sensitivity analysis was conducted by deleting the study with a NOS score of <6 to evaluate the impact of a low-quality study on the combined results. STATA software (version 15.1; Stata Corporation, University City, TX, USA) was used to create a funnel chart to assess publication bias. Publication bias of the included studies was analyzed using Egger’s test (47). A *P* value for any of those tests of ≤0.05 was indicative of the presence of bias (47).

## Supplementary Material

Supplemental file 1
