# Supplementary material for: Adjuvant β-Lactam Therapy Combined with Vancomycin or Daptomycin for Methicillin-Resistant Staphylococcus aureus Bacteremia: a Systematic Review and Meta-analysis
Source: Antimicrob Agents Chemother. 2020 Oct 20;64(11):e01377-20. doi: 10.1128/AAC.01377-20 (PMC7577142; doi:10.1128/AAC.01377-20)
Supplement: Supplemental file 1 [file AAC.01377-20-s0001.pdf]

Table S1.

Definition of outcome indicators.

**Study**	**Outcome**	**Definition**
**Casapao ,2017 [1]**	Clinical failure	Clinical failure was defined as a composite endpoint that included: (1) 30-day mortality, (2)persistent bacteremia (≥ 7 days), (3) bacteremia relapse, and/or (4) change from COMBO or STAN or addition of an anti-MRSA antibiotic to COMBO or STAN necessitated by lack of infection resolution.
**Taylor,2019[4]**	Clinical failure	Defined as 30-day mortality, persistent bacteremia, or 60-day recurrence
**Jorgensen(1),2019[3]**	Clinical failure	The primary outcome was clinical failure defined as a composite of 30-d all-cause mortality, 60-day recurrence, and persistent BSI (≥ 7 days).
**Jorgensen(2),2019[8]**	Composite clinical failure	60-day mortality and/or 60-day recurrence
**Moise,2013[9]**	Clinical failure	A failure was defined as an inadequate response to therapy, with worsening or new/recurrent signs and symptoms, a need for a change in antibiotic therapy,or a positive S.aureus blood culture reported at the end of therapy.
**Trinh,2017[5]**	Composite failure	Composite failure outcome included: 30d mortality, MRSA BSI ≥7d, and 60d recurrence.
**Truong,2018 [6]**	Clinical failure	Clinical failure included any of the following: (i) initiation of a new MRSA agent due to ongoing signs and symptoms of infection or persistent bacteremia; (ii) MRSA-related mortality, defined as positive MRSA blood cultures at the time of death or documented as the cause of death per the primary team; or (iii) readmission within 30 days of discharge for MRSA bacteremia and/or metastatic MRSA infection.
**Alosaimy,2020 [10]**	Clinical failure	Composite failure defined as: (1) 30-day mortality, (2) 60-day recurrence, or (3) persistent BSI.
Fox,2018[13]	Clinical cure	Clinical cure, defined as the improvement of signs and symptoms of bacteremia.
**Tong,2020[11]**	Clinical failure	Composite measure assessed 90 days after randomization with 4 components: (1) all-cause mortality; (2) persistent bacteremia at study day 5; (3) microbiological relapse defined as a positive blood culture for MRSA at least 72 hours after a preceding negative culture; and (4) microbiological treatment failure defined as a positive sterile site culture for MRSA at least 14 days after randomization.
**Casapao ,2017[1]**	Nephrotoxicity	Nephrotoxicity was assessed in the entire study population and was defined as a minimum of two consecutive documented increases in serum creatinine (an increase of 0.5 mg/dl or ≥ 50% increase from baseline, whichever was greater) in the absence of an alternative explanation

**Jorgensen(1),2019[3]**	Nephrotoxicity	Unclear
**Truong,2018[6]**	Acute kidney injury(AKI)	AKI, an increase in serum creatinine by ≥50% or 0.5 mg/dl, whichever was greater, from baseline in accordance with RIFLE (Risk, Injury, Failure, Loss, End-stage renal disease) criteria
**Zasowski,2019[7]**	vancomycin-associated nephrotoxicity	Vancomycin-associated nephrotoxicity defined as a serum creatinine increase of 0.5 mg/L and 50% from baseline on 2 consecutive measurements from initial vancomycin dose to 72 hours after the last dose.
**Jorgensen(2),2019[8]**	Acute kidney injury(AKI)	Acute kidney injury (AKI) was evaluated in patients not receiving hemodialysis or renal replacement therapy when DAP was started and was defined as a serum creatinine increase of ≥0.5mg/dL and 50% from baseline on two consecutive measurements while on DAP and ≤72 hours after the last dose
**Alosaimy,2020[10]**	Nephrotoxicity	Nephrotoxicity defined as serum creatinine increase of 0.5 mg/L and 50% from baseline on two consecutive measurements from initial antibiotic exposure to 72 h after the last dose
**Tong,2020[11]**	Acute kidney injury(AKI)	Defined as stage 1 or higher using modified RIFLE criteria (≥1.5-fold increase in serum creatinine; the criterion of urine output <0.5 mL/kg per hour was not included) at any time within the first 7 days or new need for renal replacement therapy (RRT) between day 1 and day 90 (participants already undergoing hemodialysis or peritoneal dialysis at randomization were excluded from this AKI end point)
**Ahmad,2020[12]**	Acute kidney injury(AKI)	Unclear
**Geriak,2019[14]**	Acute kidney injury(AKI)	Unclear

Table S2.
Study characteristics.

Author and year	Vancomycin MIC （μg/mL)			Infection site						Source control（%）
	0.25~1（%）	1（%）	1~2（%）	Skin and soft tissue infection (%)	Bone & joint (%)	Respiratory (%)	Catheter related (%)	Endocarditis (%)	CNS (%)	
Casapao,et al., 2017 [1]		STAN：1 (1–2)* COMBO ：1 (1–2) *		STAN：17.5 COMBO: 24.6	STAN：10 COMBO: 19.3	STAN：2.5 COMBO: 12.3	STAN：22.5 COMBO: 15.8	STAN：7.5 COMBO: 8.8	STAN：15 COMBO: 1.8	STAN：30 COMBO: 45.6
Davis, et al., 2016 [2]	STAN：57.1 COMBO: 64.5	STAN：28.6 COMBO: 29.0	STAN：14.3 COMBO: 6.5	STAN：21 COMBO: 23	STAN：3 COMBO: 19	STAN：10 COMBO: 0	STAN：7 COMBO: 19	STAN：0 COMBO：3	Unknown	Unknown
Jorgensen(1), et al., 2019 [3]		Unknown		skin/soft tissue (29.1%), catheter related (21.9%), osteoarticular (20.3%) and infective endocarditis (16.0%)						Unknown
Taylor, et al., 2019 [4]		Unknown		Unknown	Unknown	Unknown	Unknown	Unknown	Unknown	Unknown
Trinh, et al., 2017 [5]		Unknown		Unknown	Unknown	Unknown	Unknown	STAN：37.0 COMBO：20.0	Unknown	Unknown
Truong, et al., 2018 [6]	No	STAN：76.6 COMBO: 73.0	STAN：23.4 COMBO: 27.0	STAN：17.0 COMBO: 9.5	STAN：6.4 COMBO: 3.2	STAN：4.3 COMBO: 14.3	STAN：19.2 COMBO: 12.7	STAN：17.0 COMBO: 22.2	Unknown	STAN：63.3 COMBO: 56.7
Zasowski, et al., 2019 [7]	STAN：0.8 COMBO:	STAN：60.5 COMBO:	STAN：38.8 COMBO:	STAN：30.2 COMBO:	STAN：23.3 COMBO:	STAN：4.7 COMBO:	STAN：18.6 COMBO:	STAN：15.5 COMBO:	STAN：3.9 COMBO:	STAN：44.2 COMBO:

	0.9	57.2	41.9	16.2	10.0	31.4	21.4	23.6	1.7	34.9
Jorgensen(2), et al.,2019 [8]	STAN：24.2 COMBO: 50		STAN：75.8 COMBO: 50	STAN：15.3 COMBO: 30.6	STAN：22.9 COMBO: 37.5	Unknown	STAN：30.6 COMBO: 12.5	STAN：37.6 COMBO: 30.6	Unknown	STAN：53.5 COMBO: 59.7
Moise, et al., 2013 [9]	Unknown	Unknown	Unknown	Unknown	Unknown	Unknown	Unknown	Unknown	Unknown	Unknown
Alosaimy, et al., 2020 [10]	Unknown	Unknown	Unknown	STAN：15.7 COMBO: 12.4	STAN：26.1 COMBO: 14	STAN：5.2 COMBO: 22.1	STAN：17.9 COMBO: 17.3	STAN：18.3 COMBO: 22.0	STAN：3.9 COMBO: 1.4	STAN：52.9 COMBO: 40.5
Tong, et al., 2020 [11]	STAN：95 COMBO: 95		STAN：5 COMBO: 5	STAN：28 COMBO: 23	STAN：15 COMBO: 18	STAN：6 COMBO: 7	STAN：12 COMBO: 14	STAN：3 COMBO: 5	Unknown	STAN：80 COMBO: 73
Ahmad, et al., 2020 [12]	STAN：86.7 COMBO: 100		STAN：13.3 COMBO: 0	No	STAN：7 COMBO: 47	No	No	STAN：87 COMBO: 33	STAN：0 COMBO: 7	Unknown
Fox,et al., 2018 [13]	Unknown	Unknown	Unknown	Unknown	Unknown	Unknown	Unknown	Unknown	Unknown	Unknown
Geriak, et al., 2019 [14]	STAN：9 COMBO: 29	STAN：91 COMBO: 71	STAN：0 COMBO: 0	STAN：35 COMBO: 53	STAN：17 COMBO: 29	STAN：26 COMBO: 6	STAN：13 COMBO: 6	STAN：4 COMBO: 18	Unknown	Unknown
McCreary, et al., 2019 [15]	STAN：55 COMBO:		STAN：45 COMBO:	Unknown	Unknown	Unknown	STAN：5 COMBO:	Unknown	Unknown	STAN：40 COMBO:

57	43	5	29

*:Vancomycin MIC (mg/L), median (interquartile range,IQR)

CNS:central nervous system; COMBO: adjuvant β-lactam in combination with vancomycin in combination with vancomycin or daptomycin; STAN: standard vancomycin or daptomycin therapy alone; MIC: minimum inhibitory concentration

Table. S3. Newcastle Ottawa Risk of Bias Assessment

Study	Selection (maximum ****)				Comparability (maximum **)	Outcome (maximum ***)			Total Score (maximum 9)
	Representativeness of the exposed cohort	Selection of the non-exposed cohort	Ascertainment of exposure	Demonstration that outcome of interest was not present at start of study	Comparability of cohorts on the basis of the design or analysis	Assessment of outcome	Was follow-up long enough for outcomes to occur	Adequacy of follow up of cohorts	
**Casapao,2017**	*		*	*	*	*	*		**6**
**Taylor,2018**				*		*	*		**3**
**Zasowski,2019**	*		*	*	*	*	*		**6**
**Jorgensen(1),2019**				*	*		*		**3**
**Trinh,2017**				*	*		*		**3**
**Truong,2018**	*	*	*	*	*	*	*		**7**
**Alosaimy,2020**	*	*	*	*	*	*	*	*	**7**
**Ahmad,2020**	*	*	*	*	*	*			**6**
**Moise,2013**	*		*	*		*	*		**5**

Jorgensen(2),2019	*	*	*	*	*	*	*	7
Mccreary,2019	*		*	*	*	*	*	6
Fox,2018		*		*	*	*		4

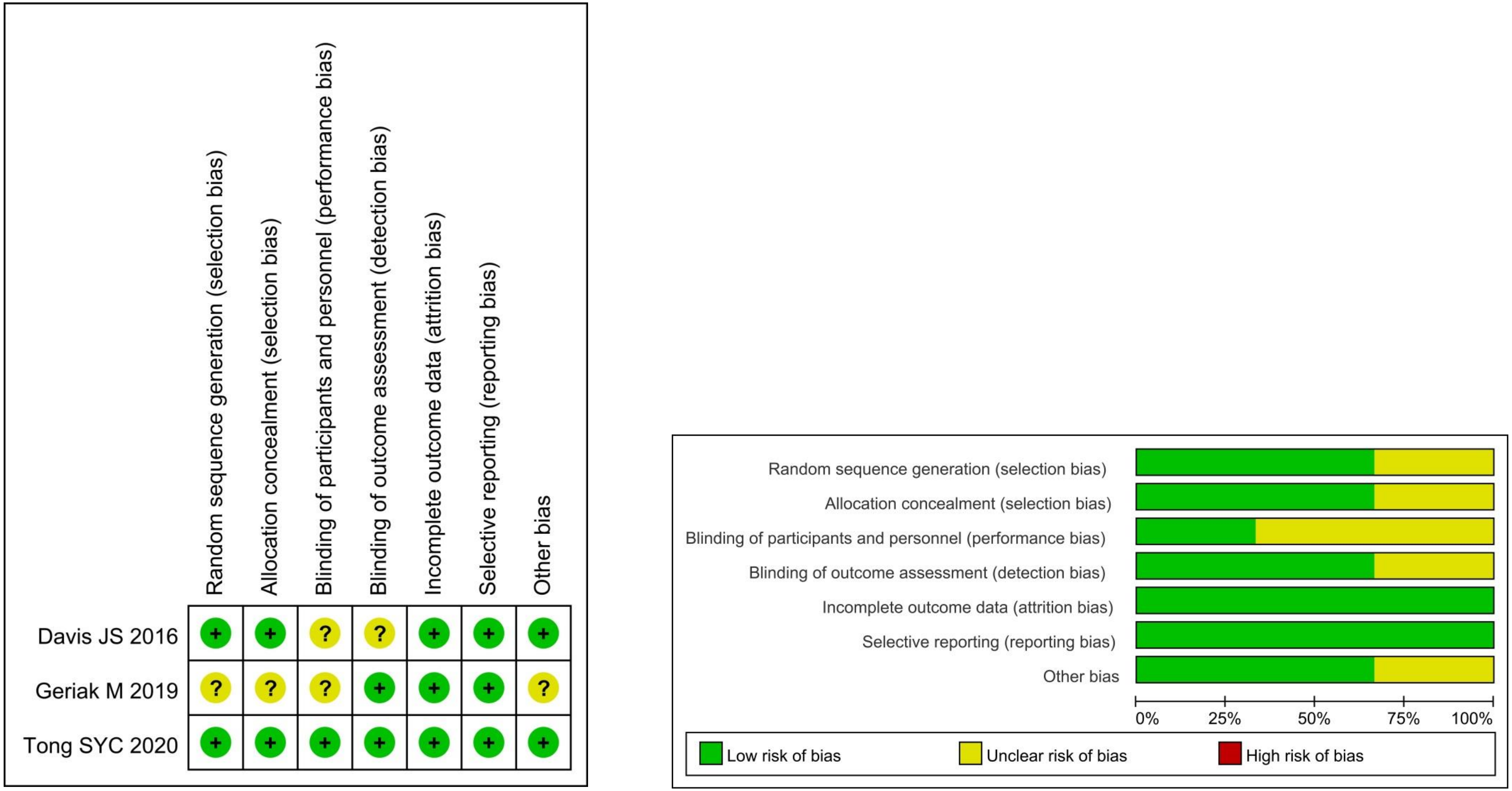

Fig. S1. Risk of bias RCT studies（left,risk of bias graph ;right,risk of bias summary）

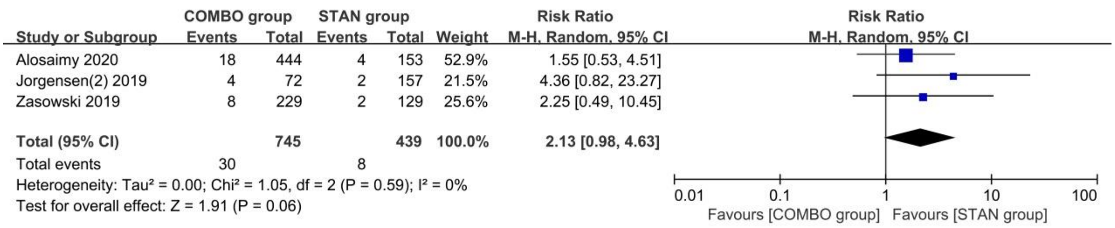

Fig.S2. Forest plot of risk ratio(RR)for *clostridium difficile* infection in patients with MRSA bacteremia

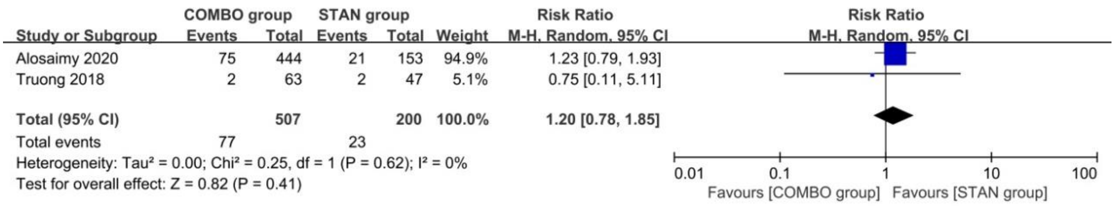

Fig. S3. Forest plot of risk ratio(RR)for thrombocytopenia in patients with MRSA bacteremia

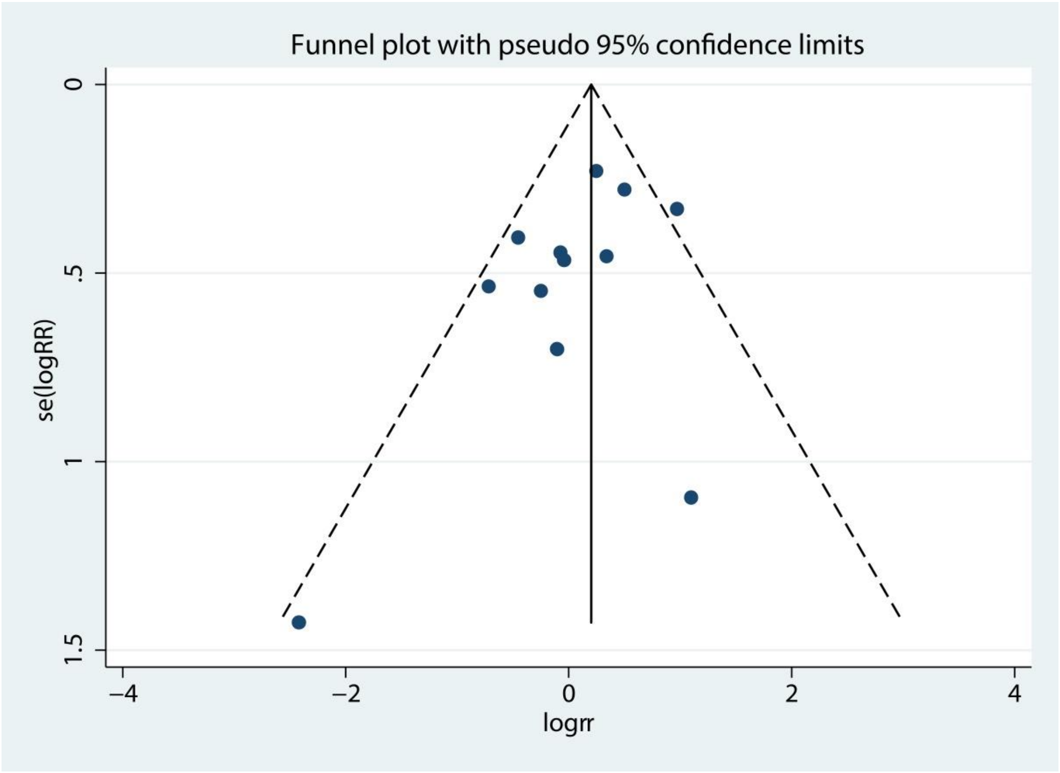

Fig. S4. Funnel chart for crude mortality indicators

REFERENCES

1. Casapao AM, Jacobs DM, Bowers DR, Beyda ND, Dilworth TJ,REACH-ID Study Group. 2017. Early Administration of Adjuvant β-Lactam Therapy in Combination with Vancomycin among Patients with Methicillin-Resistant Staphylococcus aureus Bloodstream Infection: A Retrospective, Multicenter Analysis. Pharmacotherapy 37:1347–56.
2. Davis JS, Sud A, O'Sullivan MVN, Robinson JO, Ferguson PE, Foo H, van Hal SJ, Ralph AP, Howden BP, Binks PM, Kirby A, Tong SYC; Combination Antibiotics for MEthicillin Resistant Staphylococcus aureus (CAMERA) study group; Combination Antibiotics for MEthicillin Resistant Staphylococcus aureus (CAMERA) study group, Tong S, Davis J, Binks P, Majumdar S, Ralph A, Baird R, Gordon C, Jeremiah C, Leung G, Brischetto A, Crowe A, Dakh F, Whykes K, Kirkwood M, Sud A, Menon M, Somerville L, Subedi S, Owen S, O'Sullivan M, Liu E, Zhou F, Robinson O, Coombs G, Ferguson P, Ralph A, Liu E, Pollet S, Van Hal S, Foo H, Van Hal S, Davis R.2016. Combination of Vancomycin and β-Lactam Therapy for Methicillin-Resistant Staphylococcus aureus Bacteremia: A Pilot Multicenter Randomized Controlled Trial. Clin Infect Dis 62:173–80.
3. Jorgensen S.C.J., Trinh T.D., Zasowski E.J., Alosaimy S., Melvin S., Bhatia S., Dhar S., Mynatt R.P., Pogue J.M., Rybak M.J. 2019. Combination vancomycin plus cefazolin for methicillin-resistant staphylococcus aureus bloodstream infections. Open Forum Infect Dis 6: S769.
4. Taylor D, Justo JA, Al-Hasan M, Bookstaver P, Kohn J. 2019. Risk factors for clinical failure with vancomycin therapy in mrsa bloodstream infections. Crit Care Med 47:1(Supplement 1).
5. Trinh TD, Zasowski EJ, Lagnf AM, Bhatia S, Dhar S, Mynatt R, Pogue JM, Rybak MJ.2017. Combination Vancomycin/Cefazolin (VAN/CFZ) for Methicillin-Resistant Staphylococcus aureus (MRSA) Bloodstream Infections (BSI). Open Forum Infect Dis 4: S281.
6. Truong J, Veillette JJ, Forland SC. 2018. Outcomes of Vancomycin plus a β-Lactam versus Vancomycin Only for Treatment of Methicillin-Resistant Staphylococcus aureus Bacteremia. Antimicrob Agents Chemother 62:e01554–17.
7. Zasowski EJ, Trinh TD, Atwan SM, Merzlyakova M, Langf AM, Bhatia S, Rybak MJ.2019. The Impact of Concomitant Empiric Cefepime on Patient Outcomes of Methicillin-Resistant Staphylococcus aureus Bloodstream Infections Treated With Vancomycin.Open Forum Infect

Dis 6:ofz079.

8. Jorgensen SCJ, Zasowski EJ, Trinh TD, Lagnf AM, Bhatia S, Sabagha N, Abdul-Mutakabbir JC, Alosaimy S, Mynatt RP, Davis SL, Rybak MJ.2019. Daptomycin plus beta-lactam combination therapy for methicillin-resistant Staphylococcus aureus bloodstream infections:a retrospective, comparative cohort study. Clin Infect Dis 12:ciz746.

9. Moise PA, Amodio-Groton M, Rashid M, Lamp KC, Hoffman-Roberts HL, Sakoulas G, Yoon MJ, Schweitzer S, Rastogi A. 2013. Multicenter evaluation of the clinical outcomes of daptomycin with and without concomitant β-lactams in patients with Staphylococcus aureus bacteremia and mild to moderate renal impairment. Antimicrob Agents Chemother 57:1192–200.

10. Alosaimy S, Sabagha NL, Lagnf AM, Zasowski EJ, Morrisette T, Jorgensen SCJ, Trinh TD, Mynatt RP, Rybak MJ. 2020. Monotherapy with Vancomycin or Daptomycin versus Combination Therapy with β-Lactams in the Treatment of Methicillin-Resistant Staphylococcus Aureus Bloodstream Infections: A Retrospective Cohort Analysis. Infect Dis Ther 9:325–339.

11. Tong SYC, Lye DC, Yahav D, Sud A, Robinson JO, Nelson J, Archuleta S, Roberts MA, Cass A, Paterson DL, Foo H, Paul M, Guy SD, Tramontana AR, Walls GB, McBride S, Bak N, Ghosh N, Rogers BA, Ralph AP, Davies J, Ferguson PE, Dotel R, McKew GL, Gray TJ, Holmes NE, Smith S, Warner MS, Kalimuddin S, Young BE, Runnegar N, Andresen DN, Anagnostou NA, Johnson SA, Chatfield MD, Cheng AC, Fowler VG Jr, Howden BP, Meagher N, Price DJ, van Hal SJ, O'Sullivan MVN, Davis JS; Australasian Society for Infectious Diseases Clinical Research Network.2020. Effect of Vancomycin or Daptomycin With vs Without an Antistaphylococcal β-Lactam on Mortality, Bacteremia, Relapse, or Treatment Failure in Patients With MRSA Bacteremia: A Randomized Clinical Trial. JAMA 323:527–37.

12. Ahmad O, Crawford TN, Myint T. 2020. Comparing the Outcomes of Ceftaroline Plus Vancomycin or Daptomycin Combination Therapy Versus Monotherapy in Adults with Complicated and Prolonged Methicillin-Resistant Staphylococcus Aureus Bacteremia Initially Treated with Supplemental Ceftaroline. Infect Dis Ther 9:77–87.

13. Fox M, Zeqollari K, Lee G, Pontiggia L, Byrne D, Adams J, King M, Rose L.2018. Daptomycin/Ceftaroline in Combination vs. Vancomycin for the Treatment of Methicillin-Resistant Staphylococcus aureus Bacteremia. Open Forum Infect Dis 5: S318.
14. Geriak M, Haddad F, Rizvi K, Rose W, Kullar R, LaPlante K, Yu M, Vasina L, Ouellette K, Zervos M, Nizet V, Sakoulas G. 2019. Clinical Data on Daptomycin plus Ceftaroline versus Standard of Care Monotherapy in the Treatment of Methicillin-Resistant Staphylococcus aureus Bacteremia. Antimicrob Agents Chemother 63:e02483–18.
15. McCreary EK, Kullar R, Geriak M, Zasowski EJ, Rizvi K, Schulz LT, Ouellette K, Vasina L, Haddad F, Rybak MJ, Zervos MJ, Sakoulas G, Rose WE.2019. Multicenter Cohort of Patients With Methicillin-Resistant Staphylococcus aureus Bacteremia Receiving Daptomycin Plus Ceftaroline Compared With Other MRSA Treatments. Open Forum Infect Dis 7:ofz538.
